# Remarks on Some Affections of the Spinal Column

**Published:** 1859-03

**Authors:** John H. Packard

**Affiliations:** Philadelphia


### SELECTIONS.

*Remarks on some Affections of the Spinal Column.* By John H. Packard, M. D. of Philadelphia.

There are several reasons for the paucity of our definite knowledge in regard to the diseases and injuries of the spinal column; one of the chief of which is that many of those affections are either so easily reached by ordinary remedies, or so hopeless from the outset, that their careful investigation seems a matter of no moment to those who have them in charge. Moreover, *post-mortem* examinations in these cases are attended with more difficulty, and require more laborious and careful dissection, than almost any other; nor is it always easy to obtain permission to make them.

The vertebræ, from the atlas to the coccyx inclusive, are to a great degree protected from the ordinary forms of violence; and this is due not only to their form, connections, and situation within a muscular mass, but also to the fact that in the falls which constitute the most common source of injury to them, the head and extremities are very apt to exhaust the force of the shock. But there is a variety of indirect violence not mentioned in the books, which is nevertheless of some importance; I allude to powerful pressure at or near each extremity of the chain of bones. The following case will serve to illustrate its effects upon the false vertebræ.

A coal-miner was sitting upon a large piece of coal, and bending forward to his work, when a mass was detached just over his head, and came down upon him. The force thus brought to bear was immense, and its results were in proportion; the sacrum was fractured transversely as well as longitudinally, and its lower extremity was comminuted, as was also the coccyx; the right sacro-iliac symphysis was forced open; the horizontal ramus of the pubis of each side, and the ascending ramus of the ischium of each side, were fractured. There was also a fracture of the left tibia, and a complete rupture of the urethra.

When force of this kind acts upon the true vertebræ only, it may have a very singular effect. A young man, 17 years of age, was admitted into the Pennsylvania Hospital, in September, 1855; he had been sitting upon a log, beneath a staging upon which there were a good many people, when the staging gave way. His spinal column had thus been subjected to great force at each extremity. When brought to the hospital, he was much collapsed, and suffering extreme pain; his back presented a striking prominence at about the eleventh dorsal vertebræ. The pain extended all around his body; neither the sensibility nor the motions of his lower limbs were impaired. He was laid in bed upon his right side; reaction having occurred, counter-irritation and diaphoretics were employed, and his bladder emptied once by means of a catheter. No bad symptom ensued; a week after his accident, he was able to stand up, holding by a chair; and he gradually gained strength, although the deformity of his back remained.—At the end of six weeks he was well enough to be discharged.

Now, what was the lesion in this case? The eleventh dorsal vertebra formed a very marked projection backwards; or, to speak more correctly, the spinous process of that bone constituted the apex of the angle made between the upper and lower portions of the vertebral column. There could not have been any great degree of compression of the cord, without some symptoms; but such compression would seem inevitable, if luxation had been present. Nor is it at all certain that luxation can occur in any but the cervical vertebræ; no instance is recorded in proof of such a possibility.

The supposition of a fracture was excluded by the impossibility of lessening the angular bending of the spine, by the want of crepitus, and by the rapid recovery; and besides, had such deformity been the result of fracture, injury to the cord would most certainly have been sustained.

Sir ASTLEY COOPER relates a very similar case to the above, except that in it there were two or three spinous processes broken also, and a laceration of the muscles on one side; complete recovery ensued.

It was suggested by a gentleman who saw the case of which I have given the details, that the injury was neither a fracture nor a luxation, but a squeezing out forwards of the inter-vertebral substance, the mechanism being the same as when the body of a vertebra is crushed by indirect violence. The inter vertebral fibro-cartilage is held in place by very close and strong attachments to the bones above and below; it is, moreover, confined on every side by the ligaments, and especially in front by that one which usually receives the name of *anterior common* ligament; so that such a displacement of it would seem almost impossible practically. This explanation must, therefore, be looked upon as purely theoretical, until an opportunity occurs for verifying it by discussion.

Two cases of somewhat similar deformity have come under my notice, although, as will be seen, their attendant circumstances were different: I call them similar, because they likewise concerned the eleventh dorsal vertebra, which was abnormally prominent, and because the precise nature of the lesion could not be detected. In one of them, the child was a stout and healthy girl, 4 years of age; five or six weeks previously to my seeing her, she was known to have fallen down some steps. When her mother brought her to me, she had noticed, a few days before, something peculiar in her walk, and was led to examine her, when she found a lump in her back. This lump proved to be the spine of the eleventh dorsal vertebra, projecting very slightly to the left, and maintaining perfectly its relation to the transverse processes and to the ribs; as if the lower part of the spinal column had been displaced forward *en totalité* The child walked quite feebly, and carried its shoulders a good deal backward. No effect had been produced upon the bladder or rectum. Unfortunately, this very interesting case has passed beyond my reach.

The other case was that of a girl 3½ years old, not at all healthy in appearance, and of small stature. About a year before she was brought to me, she had a very serious illness of some kind, and never perfectly recovered from it; at about the same time she fell down stairs, and to this fall her mother seemed disposed to attribute the affection of her spine. She began to be quite lame about six months afterwards — Upon examination, three months ago, the eleventh dorsal vertebra was seen to project backward to a marked degree, much as in the preceding case; and this child, like the other, walked feebly, and carried its shoulders a good deal back. There was, however, some difficulty in urination in this case, and the right lower extremity seemed shortened. An accurate investigation was almost impossible, from the extreme fretfulness of the child. A stimulating liniment was ordered, with tonics, and a simple but nutritious diet; but, as might have been predicted, no change has taken place in regard to the local affection.

Now, in these two children, fracture may be at once excluded from consideration in making a diagnosis, for obvious reasons. Luxation seems equally improbable. Might there have been a displacement, partial or complete, of the intervertebral substance, or probably a destruction of it by disease? And if so, how are we to explain the peculiar deformity, and the carrying back of the shoulders? The recent date of the first case, and the robust health of the child, excluded from my mind the idea of disease of the bones or fibro-cartilages; while in neither was there the tenderness on pressure, to say nothing of the symptoms connected with the spinal marrow, which usually accompany such affections.

To explain the cases now related, it seems to me that we must assume the possibility of some as yet undescribed lesion of the vertebral column; that neither luxation, sub-luxation, nor fracture could have existed without symptoms quite different in degree, if not in kind.—The exact nature of the lesion will probably remain obscure until an opportunity occurs for studying it by dissection.

In regard to the pathology of the spinal cord, although authors have given very positive data as the effects of injuries sustained at different parts of its extent, and although much has been written upon its diseases, there are still points which have hitherto escaped attention.

Thus, in fractures of the cervical vertebræ, a very remarkable phenomenon is sometimes observable, viz.: an intensely pungent heat of the entire surface below the seat of the injury. An instance of this is related in MORGAN'S *First Principles of Surgery*, as having occurred at St. George's Hospital, London. The temperature of the skin was 111°, while the respirations were only five or six in the minute.

DUPUYTREN mentions in connection with one of his cases, that the skin was hot; but he seems to mean merely the heat of a febrile movement, which is but slight compared with the phenomenon now under consideration. Two cases have fallen under my notice, in which it was present in a very striking degree; in one particularly, that of a man who was struck upon the back of the neck by a bale of cotton, weighing two hundred pounds, and falling from the height of four stories; the sensation of heat communicated to the hand was actually painful. Unfortunately, it was not in my power to follow up this patient, who died in about a week from the time of his accident.

How are we to explain this circumstance? Nothing is easier than to say that the rise of temperature is due to deranged innervation; but in what does the derangement consist, and why should the result be a rise, and not a fall, of temperature? It would seem as if a check were removed from some heat generating agent—possibly the sympathetic system of nerves—which, no longer controlled by the regulating influence of the cerebro spinal axis, carries on its functions to an inordinate and excessive degree. A rise of temperature similar, and perhaps equal to this, takes place in some fevers, in which the nervous system is very evidently one of the chief seats of the disordered action; but we have then another obvious reason for its occurrence, in the rapidity of the chemical changes which are going on. That this latter is at least not the sole cause of the abnormal heat in injuries of the cervical portion of the spinal marrow, is evident from the fact that respiration is so much diminished in frequency in these cases. Whether the same phenomenon is present when the dorsal or lumbar vertebræ are the ones involved, I am unable to say, but I am inclined to believe that it is not.

Paraplegia, as is well known, often exists when it is impossible to account for it; but in such cases its onset is generally slow and insidious. This rule does not, however, always hold good; sometimes the loss of power is almost instantaneous. A singular case of this kind occurred in the Pennsylvania Hospital about two years ago. The patient was a remarkably stout seaman, 22 years of age. He said that two months before his admission he had been a good deal exposed to cold and wet, while at sea, in painting the vessel; and that he felt pain and numbness in his limbs while engaged in this work. He had had no difficulty of urination; was perfectly comfortable, with a good appetite; his bowels were regular. The reflex action of his lower limbs seemed entirely set aside, and their sensibility was greatly impaired; sometimes his feet would become quite blue from the languid circulation in their vessels. He could just manage to walk, with great difficulty and uncertainty, holding by chairs, beds, or whatever he could grasp Various plans of treatment were successsively adopted—iodide of potassium, oil of turpentine, very active counter-irritation, tonics, strychnia, mercurials—but after many months of alternate improvements and relapses, he at last left the hospital in a condition very little better than when he entered it.

In another case, that of a little girl, æt. 13, at present under my charge, the affection seems to be possibly of a rheumatic origin. The child had had several attacks of rheumatism, and was under my charge for paraplegia, in the fall of 1857. After several other plans had been tried, she recovered the power of walking, while using a mixture containing phosphate of iron, in conjunction with powerful counter-irritation by means of moxas applied over the spine. I say *while using*, because it cannot be positively asserted that the improvement in her condition was altogether due to the remedies employed. Shortly afterwards she had a very severe attack of rheumatism, involving both the upper and lower extremities, and the pericardium; but after a hard struggle, she shook off this affection also. In September last, the paraplegia returned, and she is now in much the same state as when I first saw her. Her condition is in other respects good; she is fat and plump, has generally a good appetite and digestion, and sleeps well. She has not now, nor has she ever had, any difficulty either in passing or in holding her water; her bowels are very often constipated. There is some tenderness over the dorsal and lumbar spines, and some pain there, according to her account, all the time. Her lower extremities are entirely powerless, and destitute of sensibility; no reflex action occurs when the soles of her feet are tickled. No effect seems as yet to have followed from the treatment, which has consisted in the phosphate of iron, strychnia (1-20 gr. *t. d.*), and moxas to the back.

Now it is by no means difficult to conceive of rheumatism attacking the fibrous envelop of the spinal cord; but in this child's case the parts affected in the marked accessions of the disorder are the extremities; and as yet the hips have been exempt. Here is certainly something curious; a child occasionally losing all power and sensibility in her lower limbs, without any apparent cause, and liable also to very violent attacks of rheumatism. Can we assume any connection between the two? And if so, why is it that pain is so insignificant a symptom in the former, and so excruciatingly severe in the latter?—Why is it, moreover, that the two conditions come on at different times—yield to different remedies—and disappear independently of one another?

A gentleman, about 30 years of age, a lawyer by profession, had just come home from a summer trip, the last two weeks of which he had spent at the sea-shore, when he consulted me on account of extreme languor and debility, affecting especially his lower limbs. He had not taken cold, nor been fatigued in any way; nor could he assign any reason whatever for his disorder. His liver being somewhat torpid, I gave him a blue pill; and then put him upon the use of tr. ferri chlor. and quiniæ sulph. This seemed to benefit him slightly; but although his digestion was good, his bowels regular, and his urine normal, he still complained of excessive weakness, particularly in his extremities, and of numbness almost amounting to pain, in his knees. I, therefore, ordered him 1-16 of a grain of strychnia, thrice daily. As soon as he began to take this, his condition improved, until he became strong enough for horseback exercise; he then gradually left off the medicine, and has had no return of the complaint. He told me that on days when he expected to go into court, he had to omit the morning dose of strychnia, because if he took it, he could not control the starting and twitching of his legs.

I have reported these cases because they seem to me to present some points of peculiarity, and because they belong to a class of affections which are as yet obscure, although of great importance; involving, as they often do, the lifelong discomfort and even misery of the patient, and a world of annoyance and discouragement to the surgeon.—*American Journal of Medical Science.*

